# A Novel Multi-Receiver Signcryption Scheme with Complete Anonymity

**DOI:** 10.1371/journal.pone.0166173

**Published:** 2016-11-10

**Authors:** Liaojun Pang, Xuxia Yan, Huiyang Zhao, Yufei Hu, Huixian Li

**Affiliations:** 1 State Key Lab. of Integrated Services Networks, School of Life Science and Technology, Xidian Univ., Xi’an, 710071, Shaanxi, China; 2 Dept. of Comput. Sci., Wayne State University, Detroit, MI 48202, United States of America; 3 School of Computer Science and Engineering, Northwestern Polytechnical Univ., Xi’an, 710072, Shaanxi, China; King Saud University, SAUDI ARABIA

## Abstract

Anonymity, which is more and more important to multi-receiver schemes, has been taken into consideration by many researchers recently. To protect the receiver anonymity, in 2010, the first multi-receiver scheme based on the Lagrange interpolating polynomial was proposed. To ensure the sender’s anonymity, the concept of the ring signature was proposed in 2005, but afterwards, this scheme was proven to has some weakness and at the same time, a completely anonymous multi-receiver signcryption scheme is proposed. In this completely anonymous scheme, the sender anonymity is achieved by improving the ring signature, and the receiver anonymity is achieved by also using the Lagrange interpolating polynomial. Unfortunately, the Lagrange interpolation method was proven a failure to protect the anonymity of receivers, because each authorized receiver could judge whether anyone else is authorized or not. Therefore, the completely anonymous multi-receiver signcryption mentioned above can only protect the sender anonymity. In this paper, we propose a new completely anonymous multi-receiver signcryption scheme with a new polynomial technology used to replace the Lagrange interpolating polynomial, which can mix the identity information of receivers to save it as a ciphertext element and prevent the authorized receivers from verifying others. With the receiver anonymity, the proposed scheme also owns the anonymity of the sender at the same time. Meanwhile, the decryption fairness and public verification are also provided.

## Introduction

### Research backgroud

In 2000, Bellare *et al*. [1] firstly proposed the concept of multi-receiver public key encryption. In their scheme, to acquire the ciphertext which each authorized receiver can decrypt with his private key, the sender needed to repeatedly use the public key of each receiver to perform the public key encryption for the same plaintext. Although this scheme meets the requirement of the multi-receiver encryption, it is inadaptable to large-scale broadcast encryption, because its encryption computation complexity and ciphertext length are directly related to the number of the receivers. To overcome this weakness, Kurosawa [2] adopted a “randomness reuse” technique to propose a multi-receiver encryption scheme, in which the computational efficiency was improved. Later, Bellare *et al*. [3] further improved its performance. But these two schemes only concern how to improve the efficiency of multiple encryptions rather than how to reduce the number of encryptions.

Even so, these early multi-receiver schemes pointed out a new direction in the field of the information security: multi-receiver encryption, in which the sender only needs one encryption operation to send the same message for n receivers, and every authorized receiver can independently use his private key to decrypt the ciphertext, which significantly increases the efficiency comparing the early schemes [1–3]. In 2005, by introducing the idea of identity based encryption into the multi-receiver encryption, Baek *et al*. [4] proposed an efficient multi-receiver ID-based scheme, in which the sender only needed to encrypt the same message once and sent it to n selected receivers. This scheme required a linear ciphertext size in proportion to the number of the selected receivers. In 2006, Chatterjee and Sarkar [5] proposed an efficient multi-receiver ID-based scheme with sublinear ciphertext size. Later on, there appeared many great schemes [6–8] contributing to the ID-based multi-receiver encryption.

With the development of encryption, more and more researchers find that receivers need to verify the source of the message in practical applications. There are some signcryption schemes [9–12] have been proposed to advance the signcryption research. For the multi-receiver cryptography, multi-receiver signcryption gradually becomes the research focus. In 2006, the first ID-based multi-receiver signcryption scheme was presented by Duan *et al*. [13], which introduced the concept of Zheng’s signcryption [14] into multi-receiver encryption. In Duan *et al*.’s scheme, the sender can sign and encrypt the plaintext in only one operation as well as each authorized receiver can independently decrypt the ciphertext and verify the message source. Later on, many excellent multi-receiver signcryption schemes [15–21] have been proposed by researchers. However, all these early schemes did not care the privacy of participants, because the sender and receiver list, a part of the ciphertext, are required to participate in the de-signcryption process.

Recently, with the maturity of the ID-based multi-receiver signcryption, researchers have paid more attention to the anonymity of participants. Generally speaking, the anonymity includes two parts, the receiver anonymity and the sender anonymity. In 2010, Fan *et al*. [22] pointed out the importance of the receiver anonymity in ID-based multi-receiver setting and proposed a multi-receiver anonymous encryption scheme to protect anonymity of receivers with the Lagrange interpolation polynomial. In their scheme, the Lagrange interpolation polynomial is used to mix and hide the identities of the receivers to avoid exposing their information, and that seems perfect to protect the receiver anonymity. Then, several multi-receiver signcryption schemes [23–25] based on the Lagrange interpolation polynomial were proposed.

For the sender anonymity, in 2009, Lal *et al*. [26] adopted Huang *et al*.’s [27] concept of ring signature to present a multi-receiver signcryption scheme with sender anonymity. Later, based on the ring signature, several multi-receiver signcryption schemes [28–30] were proposed to protect the anonymity of the sender. However, in 2013, Pang *et al*. [31] pointed that these schemes whose sender anonymity is based on the ring signature shall suffer from the cross-comparison attack and the joint conspiracy attack. That is to say, the scope of the real sender could be narrowed down gradually with the increase of communication. Even, the identity of real sender could be uniquely determined. In order to solve this problem, Pang et al. improved the ring signature with a randomized method, which uses the public key of the sender multiplied by a random value to hide the identity of the sender. By this means, any receiver can only judge whether the ciphertext is from a reliable sender or not, rather than actually getting the real identity of the sender. Besides, the receiver anonymity with the Lagrange interpolation polynomial was provided in Pang *et al*.’s scheme [31]. So, it is a completely anonymous multi-receiver signcryption scheme.

Unfortunately, in 2012, Wang *et al*. [32] and Zhang *et al*. [33] respectively found that Fan *et al*.’s scheme fails to protect the receiver anonymity, because any authorized receiver can judge whether the others are authorized or not. This means that the authorized receivers may be attacked by other authorized receivers. Meanwhile, Wang et al. also made an improvement on Fan *et al*.’s scheme. However, in 2014, Li *et al*. [34] analyzed Wang *et al*.’s scheme and found that the Lagrange interpolation polynomial is still used to mix and hide the identities of the receivers, which is not able to really protect the receiver anonymity either. Because of the problem of Lagrange interpolation polynomial construction, any authorized receiver can judge whether other receivers is the authorized or not. Through analyses above, Pang *et al*.’s [31] completely anonymous multi-receiver signcryption scheme cannot realize the receiver anonymity. Then, it remains an open problem how to design a new multi-receiver signcryption scheme which can achieve the receiver anonymity and the sender anonymity at the same time.

### Our contribution

Aiming at the problem discussed above, in this paper, we try to find a new construction method to design a completely anonymous multi-receiver signcryption scheme cannot realize the receiver anonymity and the sender anonymity at the same time. In order to achieve the receiver anonymity, we find a new polynomial that could be used to replace the Lagrange interpolation polynomial. With the new polynomial, we can mix the identity information of receivers to save it as ciphertext element and prevent the authorized receivers from verifying the others. That is to say, attackers not only outside the system but also inside the system can be prevented in our new scheme, which can actually realize the receiver anonymity. To protect the sender anonymity, the randomized method was also used in our scheme. Hence, our scheme simultaneously has the sender anonymity and receiver anonymity, and eliminates the anonymity problem existing in the previous scheme.

### Paper organization

The rest of the paper is designed as follows. Preliminaries are given in Section 2, and Section 3 presents our new scheme. Then, we prove the security of the proposed scheme in Section 4. Section 5 gives the efficiency and performance analysis. Finally, Section 6 draws the conclusions.

## Preliminaries

In this section, we will briefly review the bilinear pairings, related problems and security assumptions on which our improved scheme is based.

### Bilinear pairings

Let *G*_1_ be a cyclic additive group of large prime order *q*, and *G*_2_ be a cyclic multiplicative group of the same order *q*. Let *P* be a generator of *G*_1_. A bilinear pairing is a map *e*: *G*_1_ × *G*_1_ → *G*_2_ and satisfies the following properties:

1. Bilinear: *e*(*aP*, *bQ*) = *e*(*P*, *Q*)^*ab*^ for all *P*, *Q* ∈ *G*_1_ and $a,b\in Z_{q}^{*}$.
2. Nondegenerate: There exist *P*, *Q* ∈ *G*_1_ such that *e*(*P*, *Q*) ≠ 1.
3. Computable: For all *P*, *Q* ∈ *G*_1_, there exists an efficient algorithm to compute *e*(*P*, *Q*).

A bilinear pairing map which satisfies the above three properties is called an admissible bilinear map.

### Problems and security assumptions

Here, we give mathematical hard problems and define the security assumptions on which our scheme is based.

(1) CDH (Computational Diffie-Hellman) problem: Given (*P*, *aP*, *bP*) ∈ *G*_1_ for some $a,b\in Z_{q}^{*}$, to compute *abP*.

**Definition 1:** The advantage of any PPT algorithm *A* in solving the Computational Diffie-Hellman (CDH) problem is defined as:

$$\begin{array}{c} Adv_{A}^{CDH}=Pr\left[A\left(P,aP,bP\right)=abP\right] \end{array}$$ (1)

**CDH assumption:** For any PPT algorithm *A*, $Adv_{A}^{CDH}$ is negligible.

(2) DBDH (Decision Bilinear Diffie-Hellman) problem: Given (*P*, *aP*, *bP*, *cP*) ∈ *G*_1_ for unknown $a,b,c\in Z_{q}^{*}$, and *R* ∈ *G*_2_, to decide whether *e*(*P*, *P*)^*abc*^ = *R*.

**Definition 2:** The advantage of any PPT algorithm *A* in solving the DBDH (Decision Bilinear Diffie-Hellman) problem is defined as:

$$\begin{array}{c} Adv_{A}^{DBDH}=\left|Pr\left[A\left(P,aP,bP,cP,e{\left(P,P\right)}^{abc}\right)=1\right]-Pr\left[A\left(P,aP,bP,cP,R\right)=1\right]\right| \end{array}$$ (2)

**DBDH assumption:** For any PPT algorithm *A*, $Adv_{A}^{DBDH}$ is negligible.

(3) Gap-BDH (Gap Bilinear Diffie-Hellman) problem: Given (*P*, *aP*, *bP*, *cP*) ∈ *G*_1_ for unknown $a,b,c\in Z_{q}^{*}$, to compute *e*(*P*, *P*)^*abc*^ ∈ *G*_2_ with the help of the DBDH (Decision Bilinear Diffie-Hellman) oracle.

**Definition 3:** The advantage of any PPT algorithm A in solving the Gap-BDH (Gap Bilinear Diffie-Hellman) problem is defined as:

$$\begin{array}{c} Adv_{A}^{Gap-BDH}=Pr\left[A\left(P,aP,bP,cP\right)=e{\left(P,P\right)}^{abc}\right]>\varepsilon \end{array}$$ (3)

**Gap-BDH assumption:** For any PPT algorithm *A*, $Adv_{A}^{Gap-BDH}$ is negligible.

### Security models

We shall give the security models for confidentiality, unforgeability and anonymity in Definitions 4-6, respectively.

**Definition 4:** IND-sMIBSC-CCA (indistinguishability of ciphertexts under selective multi-ID, chosen ciphertext attack) [13].

Suppose that there is a polynomial-time attacker named *A* and an anonymous ID-based multi-receiver signcryption algorithm named *Π*. *A* plays a game with a Challenger *B* as follows:

**Setup:** Challenger *B* performs this algorithm to generate master key *s* and public parameters params. Then *B* shall send the params to *A* but keep *s* secret. After receiving the parameter, *A* outputs target multiple identities $L^{*}=\left\{ID_{1}^{*},ID_{2}^{*},\cdots ,ID_{n}^{*}\right\}$.

**Phase 1:** Challenger *B* shall answer a number of different queries from adversary *A* in an adaptive manner as follows:

**Key extract query:** Queried about an identity ID that *A* pretends to be, *B* shall run the Key extract algorithm to get *D* = *Extract*(*parems*, *s*, *ID*).

**Anony-signcrypt query:** Adversary *A* runs the Anony-signcrypt algorithm to get the ciphertext *C* = *Anony* − *signcrypt*(*parems*, *M*, *L*, *D*_*S*_), where *M* is the target plaintext chosen by adversary *A*, *L* = {*ID*_1_, *ID*_2_, ⋯, *ID*_*n*_} is the set of the receiver identity, *ID*_*S*_ is the identity chosen by *B* and *D*_*S*_ is the corresponding private key.

**De-signcrypt query:** Adversary *A* shall send *B*(*C*, *ID*_*j*_) where *C* is the ciphertext produced by adversary *A*, *ID*_*j*_ is the identity chosen by *B* and *ID*_*j*_ ∈ L*. $L^{*}=\left\{ID_{1}^{*},ID_{2}^{*},\cdots ,ID_{n}^{*}\right\}$ is the target multiple identities chosen by *A*. Then *B* shall perform the De-signcryption algorithm to get the plaintext $M=De-signcrypt(C^{*},params,D_{i}^{*})$. If *M* is valid, *B* returns it to *A*. Otherwise, returns “failure”.

**Challenge:** Adversary *A* shall first choose target plaintext pair(*M*_0_, *M*_1_) and pretend a sender *ID*_*S*_. When receiving the target plaintext and the private key *D*_*S*_, the challenger *B* randomly chooses *β* ∈ {0, 1} and signcrypts the message *M*_*β*_ to generate the ciphertext C* = *Anony* − *signcrypt*(*params*, *M*_*β*_, L*, *D*_*S*_). Then, the challenger *B* returns C* to *A*.

**Phase 2:** *A* shall query challenge *B* like Phase 1. Note that *A* cannot query the information of $\left(ID_{1}^{*},ID_{2}^{*},\cdots ,ID_{n}^{*}\right)$ in the Key extract query and C* in De-signcrypt query.

**Guess:** *A* guesses *β*′ ∈ {0, 1} and outputs it. If *β* = *β*′, *A* wins the IND-sMIBSC-CCA game. Otherwise, returns “failure”.

*A*’s guessing advantage is defined as follows:

$$\begin{array}{c} Adv_{\Pi }^{IND-sMIBSC-CCA}=\left|Pr\left[\beta =\beta^{{}^{\prime }}\right]-1/2\right| \end{array}$$

The scheme *Π* is said to be (*t*, *ε*)-IND-sMIBSC-CCA secure, if for any IND-sMIBSC-CCA attacker *A*, its guessing advantage is less than *ε* within polynomial running time *t*.

**Definition 5:** SUF-MIBSC-CMA (strong existential unforgeability under selective multi-ID, chosen message attack) [13].

Suppose that there is a forger named *F* and an anonymous ID-based multi-receiver signcryption algorithm named *Π*. *F* plays a game with a challenger *B* as follows:

**Setup:** Challenger *B* performs this algorithm to generate master key *s* and public parameters params. Then *B* shall send the params to *A* but keep *s* secret. After receiving the parameter, *F* outputs target multiple identities $L^{*}=\left\{ID_{1}^{*},ID_{2}^{*},\cdots ,ID_{n}^{*}\right\}$.

**Attack:** The forger *F* may make some queries to the challenger *B* as phase 1 in Definition 4.

**Forgery:** Forger *F* shall output a ciphertext C* and a set of identities $L^{*}=\left\{ID_{1}^{*},ID_{2}^{*},\cdots ,ID_{n}^{*}\right\}$. If C* can be decrypted correctly by every receiver $ID_{i}^{*}$ where *i* ∈ {1, 2, ⋯, *n*} in the set L*, then verify the source of the sender, C* is valid and *F* wins the game.

But the forger *F* cannot perform Key extract query to $ID_{i}^{*}$ and C* cannot generated by Anony-signcrypt algorithm here.

The scheme *Π* is said to be (*t*, *ε*)-SUF-MIBSC-CMA secure, if for any SUF-MIBSC-CMA forger *F*, its guessing advantage is less than *ε* within polynomial running time *t*.

**Definition 6:** ANON-IND-sMID-CCA (anonymous indistinguishability of signcryption under selective multi-ID, chosen ciphertext attack) [25].

Suppose that there is a polynomial-time attacker named *A* and an anonymous ID-based multi-receiver signcryption algorithm named *Π*. In order to get the identity of anonymous receivers, *A* plays a game with a challenger *B* as follows:

**Setup:** Challenger *B* performs this algorithm to generate master key *s* and public parameters params. Then *B* shall send the params to *A* but keep *s* secret. After receiving the parameter, *A* choses target identities $\left(ID_{1}^{*},ID_{2}^{*}\right)$.

**Phase 1:** Challenger *B* shall answer the Key extract query and De-signcryption query from adversary *A* as follows:

**Key extract query:** Queried about an identity *ID*_*j*_ that *A* pretends to be, where $ID_{j}\neq \left(ID_{1}^{*},ID_{2}^{*}\right)$, *B* shall run the Extract algorithm to get *D*_*j*_ = *Extract*(*parems*, *s*, *ID*_*j*_).

**De-signcrypt query:** Adversary *A* shall send $B(C^{*},ID_{i}^{*})$ where *i* ∈ {1, 2} to *B*. Then *B* shall perform the De-signcryption algorithm to get the plaintext $M=De-signcrypt(C^{*},params,D_{i}^{*})$. If *M* is valid, *B* returns it to *A*. Otherwise, returns “failure”.

**Challenge:** Adversary *A* shall first choose target plaintext M* and the identities $\left\{ID_{3}^{*},ID_{4}^{*},\cdots ,ID_{n}^{*}\right\}$, where *n* ≥ 3. Then *B* shall execute the signcryption algorithm to generate the ciphertext $C^{*}=Anony-signcrypt\left(params,M^{*},\left(ID_{\beta }^{*},ID_{3}^{*},ID_{4}^{*},\cdots ,ID_{n}^{*}\right),D_{j}\right)$. Then, the challenger *B* returns C* to *A*.

**Phase 2:** A shall query challenge *B* like Phase 1 without querying for C* in De-signcrypt query the information of $\left(ID_{1}^{*},ID_{2}^{*}\right)$ in the Key extract query.

**Guess:** *A* guesses *β*′ ∈ {1, 2} and outputs it. If *β* = *β*′, *A* wins the ANON-IND-sMID-CCA game.

*A*’s guessing advantage is defined as follows:

$$\begin{array}{c} Adv_{\Pi }^{ANON-IND-sMID-CCA}\left(A\right)=\left|Pr\left[\beta =\beta^{{}^{\prime }}\right]-1/2\right| \end{array}$$

The scheme *Π* is said to be ANON-IND-sMID-CCA secure, if for any ANON-IND-sMID-CCA attacker *A*, its guessing advantage is less than *ε* within polynomial running time *t*.

## The proposed scheme

In this section, we will present our scheme, which includes four algorithms: Setup, Key extract, Anony-signcrypt, and De-signcrypt algorithms. Detailed description is as follows:

### Setup algorithm

Here, PKG shall execute the following process:

1. PKG chooses a prime order *q*(*q* ≥ 2^*l*^, *l* is a long integer), and then chooses *G*_1_ (an additive group) and *G*_2_ (a multiplicative group) with the same order *q*. Then it randomly picks a generator *P* of *G*_1_, and constructs a bilinear mapping *e*: *G*_1_ × *G*_1_ → *G*_2_. PKG keeps the master key *s* secret, which is picked up from $Z_{q}^{*}$. Select some integer *w*. Set *P*_*pub*_ = *sP* ∈ *G*_1_ as the system public key. The symmetric encryption and decryption are denoted as *E*_*k*_() and *D*_*k*_() where *k* is the key.
2. PKG constructs five cryptographic hash functions: *H*_1_: {0, 1}* → *G*_1_; $H_{2}:G_{2}⟶Z_{q}^{*}$; $H_{3}:Z_{q}^{*}⟶{\left\{0,1\right\}}^{w}$; *H*_4_: {0, 1}^*w*^ → {0, 1}^|*M*|^; $H_{5}:G_{1}\times G_{1}\times {\left\{0,1\right\}}^{w}\times Z_{q}^{*}\times Z_{q}^{*}\times \cdots \times Z_{q}^{*}⟶Z_{q}^{*}$.
3. PKG publishes the system parameters *params* = {*q*, *G*_1_, *G*_2_, *e*, *P*, *P*_*pub*_, *H*_1_, *H*_2_, *H*_3_, *H*_4_, *H*_5_, *E*_*k*_(), *D*_*k*_()}.

### Key extract algorithm

PKG shall execute this algorithm to generate *ID*_*i*_’s private key with *s*, params and an identity *ID*_*i*_ ∈ {0, 1}*. Then, PKG shall also return *ID*_*i*_’s private key. That means *ID*_*i*_ has registered himself at PKG:

1. Compute *ID*_*i*_’s public key *Q*_*i*_ = *H*_1_(*ID*_*i*_).
2. Compute *ID*_*i*_’s public key *D*_*i*_ = *sH*_1_(*ID*_*i*_) = *sQ*_*i*_.

### Anony-signcrypt algorithm

This algorithm is executed by the sender. Obtaining his private key *D*_*S*_ and params, the sender *ID*_*S*_ shall choose *n* receivers with identities *ID*_1_, *ID*_2_, ⋯, *ID*_*n*_ and encrypt the plaintext *M* to generate the ciphertext *C*:

1. The sender firstly pick up two random integers $\gamma ,\alpha \in Z_{q}^{*}$ and a bit string *δ* ∈ {0, 1}^*w*^, and then compute *Y* = *rQ*_*S*_, *U* = *rP*, *X* = *αY* and *J* = *rP*_*pub*_, where *Q*_*S*_ is the public key of *ID*_*S*_.
2. The sender computes *υ*_*i*_ = *H*_2_(*e*(*Q*_*i*_, *J*), where *Q*_*i*_ = *H*_1_(*ID*_*i*_).
3. The sender chooses a random $p\in Z_{q}^{*}$ and constructs a polynomial *f*(*x*) with degree *n* as follows:
  $$\begin{array}{ccc} f(x) & = & \prod_{i=1}^{n}\left(x-\upsilon_{i}\right)+p\left(mod\;q\right)\\ & = & a_{0}+a_{1}x+\cdots +a_{n-1}x^{n-1}+x^{n} \end{array}$$
4. Compute *V* = *δ* ⨁ *H*_3_(*p*), *Z* = *E*_*H*_4_(*δ*)_(*M*) and *h* = *H*_5_(*X*, *U*, *Z*, *V*, *a*_0_, *a*_1_, ⋯, *a*_*n*−1_), and then compute *W* = (*α* + *h*)⋅*rD*_*S*_, where *D*_*S*_ is the private key of *ID*_*S*_.
5. Generate the ciphertext: *C* = 〈*Y*, *U*, *Z*, *V*, *W*, *a*_0_, *a*_1_, ⋯, *a*_*n*−1_〉.

### De-signcrypt algorithm

This algorithm is executed by the receiver. With params, *C* = 〈*Y*, *U*, *Z*, *V*, *W*, *a*_0_, *a*_1_, ⋯, *a*_*n*−1_〉, the receiver’s identity *ID*_*i*_ and his private key *D*_*i*_ as input, the receiver *ID*_*i*_ has the ability to decrypt *C* as follows:

1. Compute *h* = *H*_5_(*X*, *U*, *Z*, *V*, *a*_0_, *a*_1_, ⋯, *a*_*n*−1_).
2. Public verification: The one who has not registered shall execute this step. The participant who has registered shall jump to the judgment algorithm without the verification.
  If the equation *e*(*W*, *P*) = *e*(*X* + *hY*, *P*_*pub*_) holds, that is to say, the ciphertext is valid. Otherwise, the ciphertext has been damaged or it is invalid.
3. Judgment: The registered participants shall execute this step before the decryption process.
  If the equation *e*(*W*, *Q*_*i*_) = *e*(*X* + *hY*, *D*_*i*_) holds, *ID*_*i*_ is one of the receivers chosen by the sender and the ciphertext is valid. Otherwise, the receiver shall quit the decryption process.
4. Compute $\upsilon_{i}^{{}^{\prime }}=H_{2}\left(e\left(D_{i},U\right)\right)$ and $p=f(\upsilon_{i}^{{}^{\prime }})$.
5. Compute *δ* = *V* ⨁ *H*_3_(*p*) and *K* = *H*_4_(*δ*).
6. Decryption: *M*′ = *D*_*H*_4_(*δ*)_(*Z*).

Every receiver who gets the ciphertext can verify the validity of the message by the public verification or judge if he is authorized by the judgment algorithm. Then, if necessary, he can decrypt the ciphertext.

## Correctness and security analysis

### Correctness analysis

Here, we show the correctness of the proposed scheme by stating Theorems 1-3.

**Theorem 1:** The public verification of the proposed scheme is correct.

**Proof:** Whether the equation *e*(*W*, *P*) = *e*(*X* + *hY*, *P*_*pub*_) holds is used to perform the public verification because of the following:

$$\begin{array}{ccc} e(W,P) & = & e(\left(\alpha +h\right)\cdot rD_{S},P)\\ & = & e(\left(\alpha +h\right)\cdot rQ_{S},sP)\\ & = & e(\alpha Y+hY,sP)\\ & = & e(X+hY,P_{pub}) \end{array}$$

**Theorem 2:** The judgement of the proposed scheme is correct.

**Proof:** Whether the equation *e*(*W*, *Q*_*i*_) = *e*(*X* + *hY*, *D*_*i*_) holds is used to perform the judgement because of the following:

$$\begin{array}{ccc} e(W,Q_{i}) & = & e(\left(\alpha +h\right)\cdot rD_{S},Q_{i})\\ & = & e(\left(\alpha +h\right)\cdot rQ_{S},sQ_{i})\\ & = & e(\alpha Y+hY,D_{i})\\ & = & e(X+hY,D_{i}) \end{array}$$

**Theorem 3:** The decryption of the proposed scheme is correct.

**Proof:** The decryption of the proposed scheme is correct because of the following:

$$\begin{array}{ccc} \upsilon_{i}^{\prime } & = & H_{2}\left(e\left(D_{i},U\right)\right)\\ & = & H_{2}\left(e\left(sQ_{i},U\right)\right)\\ & = & H_{2}\left(e\left(Q_{i},rsP_{pub}\right)\right)\\ & = & H_{2}\left(e\left(Q_{i},J\right)\right)\\ & = & \upsilon_{i} \end{array}$$

### Security analysis

Here, we shall prove that the proposed multi-receiver signcryption scheme is secure against the IND-sMIBSC-CCA, SUF-MIBSC-CMA and ANON-IND-sMID-CCA attacks defined in Section 2.3, which respectively shows the confidentiality, unforgeability, and anonymity.

**Theorem 4:** If an IND-sMIBSC-CCA attacker *A* has a non-negligible advantage *ε* to win the game defined in Definition 4 within running time *t*, then the DBDH problem can be solved by the challenger B in running time *t*′ ≤ *t* with a non-negligible advantage *ε*′ ≥ *ε* − *nq*_*d*_/2^*k*^, where attacker *A* asks *q*_*e*_ queries to the Key extract query, *q*_*s*_ queries to the Anony-signcrypt query, and *q*_*d*_ queries to the De-signcrypt query. (*q*_*H*_1__, *q*_*H*_2__, *q*_*H*_3__, *q*_*H*_4__, *q*_*H*_5__) denote the number of queries to the hash functions *H*_1_, *H*_2_, *H*_3_, *H*_4_, *H*_5_, respectively.

**Proof:** An instance (*P*, *aP*, *bP*, *cP*) of the DBDH problem is given to simulate the game defined in Definition 4, and *A* denotes attacker, *B* denotes challenger. Suppose that *A* has a non-negligible advantage *ε* to break the IND-sMIBSC-CCA model, and *B* solves the instance of DBDH problem by interacting with *A*. There are five oracles *H*_1_, *H*_2_, *H*_3_, *H*_4_ and *H*_5_ to simulate the system for *B*. *A* can queries PPT times to the oracles. *B* executes and answers each phase of the IND-sMIBSC-CCA game as follows:

**Setup:** The challenger *B* sets *Q* = *aP* and *P*_*pub*_ = *bP*. Then, *B* sends 〈*G*_1_, *G*_2_, *q*, *e*, *P*, *P*_*pub*_, *H*_1_, *H*_2_, *H*_3_, *H*_4_, *H*_5_, *E*_*k*_, *D*_*k*_〉 to *A* as the public parameters. When receiving the parameter, *A* outputs target multiple identities $\left(ID_{1}^{*},ID_{2}^{*},\cdots ,ID_{n}^{*}\right)$.

**Phase 1:** *A* proposes queries as follows to *B*.

Assume that the hash functions *H*_*i*_(*i* = 1, 2, 3, 4, 5) are random oracles controlled by the challenger *B*. For the attacker *A*’s hash queries, the challenger *B* uses list *L*_*i*_(*i* = 1, 2, 3, 4, 5) to record the results of hash functions *H*_*i*_(*i* = 1, 2, 3, 4, 5), respectively.

***H*_1_-query:**

1. If $ID_{j}\neq ID_{i}^{*},i\in \left\{1,2,\cdots ,n\right\}$, calculate *Q*_*j*_ = *l*_*j*_ *P*; otherwise, calculate *Q*_*j*_ = *l*_*j*_ *Q*, where *l*_*j*_ is an integer.
2. Put it into *H*_1_-list when no (*ID*_*j*_, *l*_*j*_, *Q*_*j*_) exists in *H*_1_-list.
3. *B* returns *Q*_*j*_.

***H*_2_-query:** The challenger *B* examines if (*P*, *Q*_*i*_, *P*_*pub*_, *cP*, *X*_*j*_) uses the DBDH oracle for *i* ∈ [1, *q*_*H*_2__] when he is queried with *X*_*j*_ ∈ *G*_2_ for some *j* = [1, *q*_*H*_2__]. If it exists, *B* shall terminate the game for *e*(*P*, *P*)^*abc*^ equals ${\left(X_{j}\right)}^{l_{i}^{-1}}$. Otherwise, *B* picks a value $x_{j}\in Z_{q}^{*}$ at random and puts a tuple (*X*_*j*_, *x*_*j*_) into the list *L*_2_. Then, the challenger *B* returns *x*_*j*_ to the adversary *A*.

***H*_3_-query:** As an integer *p*_*j*_ is sent to the *H*_3_ oracle where *j* ∈ [1, *q*_*H*_3__], *B* shall pick a string *w*_*j*_ ∈ {0, 1}^*w*^ at random and puts the tuple (*p*_*j*_, *w*_*j*_) into the list *L*_3_. Then, the string *w*_*j*_ is returned to *A* by the challenger *B*.

***H*_4_-query:** When querying for the string *δ*_*j*_ ∈ {0, 1}^*w*^ where *j* ∈ [1, *q*_*H*_4__], *B* shall pick a string *z*_*j*_ ∈ {0, 1}^|*M*|^ at random and puts the tuple (*δ*_*j*_, *z*_*j*_) into the list *L*_4_. Then, the challenger *B* returns the bit string *z*_*j*_ to the attacker *A*.

***H*_5_-query:** Receiving the tuple 〈*X*_*j*_, *U*_*j*_, *Z*_*j*_, *V*_*j*_, *a*_*j*_0__, *a*_*j*_1__, ⋯, *a*_*j*_*n*−1__〉 where *j* ∈ [1, *q*_*H*_5__], *B* picks a value *h*_*j*_ in $Z_{q}^{*}$ at random and puts the tuple 〈*X*_*j*_, *U*_*j*_, *Z*_*j*_, *V*_*j*_, *a*_*j*_0__, *a*_*j*_1__, ⋯, *a*_*j*_*n*−1__, *h*_*j*_〉 into the list *L*_5_. Then, *B* returns *h*_*j*_.

**Key extract query:** *A* chooses an identity $ID_{j}\neq ID_{i}^{*}$ where *i* ∈ {1, 2} and sends it to challenger *B*, then *B* scans the list *L*_1_ to find if there is the tuple (*ID*_*j*_, *l*_*j*_, *Q*_*j*_) in *L*_1_. If it was, *B* shall calculate *D*_*j*_ = *l*_*j*_ *P*_*pub*_(= *l*_*j*_ *bP* = *bQ*_*j*_). Otherwise, the challenger *B* selects a $l_{j}\in Z_{q}^{*}$ at random, and calculates *Q*_*j*_ = *l*_*j*_ *P* as well as *Dj* = *l*_*j*_ *P*_*pub*_. At the same time, the challenger *B* puts a tuple (*ID*_*j*_, *l*_*j*_, *Q*_*j*_) into the list *L*_1_. Finally, *B* sends *D*_*j*_ back to the attacker *A*.

**Anony-signcrypt query:** When receiving the anonymous signcryption query with (*M*, *ID*_*S*_, *L*) from *A*, *B* checks whether there exist $ID_{S}\neq ID_{i}^{*}\left(i=1,2,\cdots ,n\right)$. If $ID_{S}\neq ID_{i}^{*}\left(i=1,2,\cdots ,n\right)$, *B* can get the private key of *ID*_*S*_ from Key extract query. Then, *A* can get ciphertext *C* from Anony-signcrypt query. Otherwise, perform the following tasks:

1. Select $\gamma ,\alpha \in Z_{q}^{*}$ and *δ* ∈ {0, 1}^*w*^ at random, then compute *Y* = *γl*_*S*_ *P*, *U* = *γP*, *X* = *αY*, *J* = *γP*_*pub*_.
2. Compute *υ*_*i*_ = *H*_2_(*e*(*Q*_*i*_, *J*)), where *Q*_*i*_ = *H*_1_(*ID*_*i*_) is the public key of the receiver.
3. Choose $p\in Z_{q}^{*}$ at random and structure a polynomial *f*(*x*) with degree *n* as follows:
  $$\begin{array}{ccc} f(x) & = & \prod_{i=1}^{n}\left(x-\upsilon_{i}\right)+p\left(mod\;q\right)\\ & = & a_{0}+a_{1}x+\cdots +a_{n-1}x^{n-1}+x^{n}. \end{array}$$
4. Compute *V* = *δ* ⨁ *H*_3_(*p*), *Z* = *E*_*H*_4_(*δ*)_(*M*) and *h* = *H*_5_(*X*, *U*, *Z*, *V*, *a*_0_, *a*_1_, ⋯, *a*_*n*−1_), and then compute *W* = (*α* + *h*)*l*_*S*_ *P*_*pub*_.
5. Generate the ciphertext: *C* = 〈*Y*, *U*, *X*, *Z*, *V*, *W*, *a*_0_, *a*_1_, ⋯, *a*_*n*−1_〉.

**De-signcrypt query:** The attacker *A* queries *B* and send $B(C_{j},ID_{i}^{*})$ where *i* ∈ {1, 2} and *C*_*j*_ = 〈*Y*_*j*_, *U*_*j*_, *X*_*j*_, *Z*_*j*_, *V*_*j*_, *W*_*j*_, *a*_*j*_0__, *a*_*j*_1__, ⋯, *a*_*j*_*n*−1__〉 When receiving the decryption query, *B* executes the following steps:

1. Check the list *L*_5_ to find the tuple 〈*X*_*j*_, *U*_*j*_, *Z*_*j*_, *V*_*j*_, *a*_*j*_0__, *a*_*j*_1__, ⋯, *a*_*j*_*n*−1__. If it was found, *B* can get (*Z*_*j*_, *V*_*j*_) from *L*_5_. Otherwise, *B* returns “failure”.
2. Construct the polynomial *f*(*x*) = *a*_*j*_0__ + *a*_*j*_1__ *x* + ⋯+*a*_*j*_*n*−1__ *x*^*n*−1^+*x*^*n*^.
3. Searching the tuple (*ID*_*j*_, *l*_*j*_, *Q*_*j*_) in the list *L*_1_.
4. For *l* = 1, 2, ⋯, *q*_*H*_2__, perform as follows:
  - Search the tuple (*X*_*l*_, *x*_*l*_) from the list *L*_2_.
  - Examine whether (*P*, *Q*_*i*_, *P*_*pub*_, *U*_*j*_, *X*_*j*_) uses the DBDH oracle by verifying the equation *e*(*P*, *P*)^*l*_*j*_*bγ*^ = *X*_*j*_.
  - If the step above is true, calculate *p*_*l*_ = *f*(*x*_*l*_), $\delta_{j}^{\prime }=V_{j}⨁H_{3}\left(p_{i}\right)$, and $M_{j}=D_{H_{4}}\left(\delta_{j}^{\prime }\right)\left(Z_{j}\right)$.
5. Test whether the equation *e*(*W*_*j*_, *P*) = *e*(*X*_*j*_ + *h*_*j*_ *Y*_*j*_, *P*_*pub*_) or the equation *e*(*W*_*j*_, *Q*_*i*_) = *e*(*X*_*j*_ + *h*_*j*_ *Y*_*j*_, *D*_*i*_) holds where *h*_*j*_ = *H*_5_(*X*_*j*_, *U*_*j*_, *Z*_*j*_, *V*_*j*_, *a*_*j*_0__, *a*_*j*_1__, ⋯, *a*_*j*_*n*−1__). If it holds, then return *M*_*j*_ to *A*.
6. Otherwise, *B* sends “failure” to *A*, which means that there is not a valid ciphertext generated following the proposed scheme.

**Challenge:** *A* outputs a target plaintext pair (*M*_0_, *M*_1_) and a private key *D*_*S*_. Upon receiving (*M*_0_, *M*_1_) and *D*_*S*_, the challenger *B* randomly chooses *β* ∈ {0, 1} and signcrypts the message *M*_*β*_. *B* finally creates a target ciphertext C* = 〈*Y*, *U*, *X*, *Z*, *V*, *a*_0_, *a*_1_, ⋯, *a*_*n*−1_〉, where *Y* = *γl*_*S*_ *P*, *U* = *γP*, *X* = *αY*, *Z* = *E*_*H*_4_(*δ*)_(*M*), *V* = *δ* ⨁ *H*_3_(*p*) and *W* = (*α* + *h*)*l*_*S*_ *P*_*pub*_, then returns C*to *A*.

**Phase 2:** *A* shall query challenge *B* like Phase 1. Note that *A* cannot query the information of $\left(ID_{1}^{*},ID_{2}^{*},\cdots ,ID_{n}^{*}\right)$ in the Key extract query and C* in De-signcrypt query.

**Guess:** The attacker *A* gives its guess *β*′ ∈ {0, 1}. If *β*′ = *β*, *B* wins the game because the equation *Ψ* = *e*(*P*_*pub*_, *P*_1_)^*α*^ = *e*(*P*, *P*)^*abc*^ holds. Otherwise, *B* outputs “failure”.

According the above discussion, we can get the advantage of *B* as following equation. For *q*_*d*_ times De-signcrypt query, the probability for *B* to reject the valid plaintext is less than *nq*_*d*_/2^*k*^. So, if *A* wins the game, *B*’s advantage is

$$\begin{array}{ccc} \varepsilon^{\prime } & = & |Pr\left[A\left(aP,bP,cP,w\right)=1\right]-Pr\left[A\left(aP,bP,cP,e{\left(P,P\right)}^{abc}\right)=1\right]|\\ & \geq & |\varepsilon +1/2-nq_{d}/2^{k}-1/2|\\ & = & \varepsilon -nq_{d}/2^{k} \end{array}$$

**Theorem 5:** If a SUF-sMIBSC-CMA forger *F* has a non-negligible advantage *ε* to win the game defined in Definition 5 within time *t*, then the challenger *B* can solve the CDH problem with an advantage *ε*′ ≥ *ε* − *q*_*s*_/2^*k*^ in running time *t*′ ≤ *t*, where the forger *F* can ask at most *q*_*e*_ Key extract queries, *q*_*s*_ Anony-signcrypt queries and *q*_*d*_ De-signcrypt queries. (*q*_*H*_1__, *q*_*H*_2__, *q*_*H*_3__, *q*_*H*_4__, *q*_*H*_5__) denote the number of queries to the hash functions *H*_1_, *H*_2_, *H*_3_, *H*_4_, *H*_5_, respectively.

**Proof:** An instance (*P*, *aP*, *bP*) of the CDH problem is given to simulate the game defined in Definition 5, and *F* denotes the forger, *B* denotes challenger. Suppose that *F* has a non-negligible advantage *ε* to break the SUF-sMIBSC-CMA model, and *B* solves the instance of CDH problem by interacting with *F*. There are five oracles *H*_1_, *H*_2_, *H*_3_, *H*_4_ and *H*_5_ to simulate the system for *B*. *F* can queries PPT times to the oracles. *B* executes and answers each phase of this game as follows:

**Setup:** The challenger *B* sets *P*_*pub*_ = *bP* and sends 〈*G*_1_, *G*_2_, *q*, *e*, *P*, *P*_*pub*_, *H*_1_, *H*_2_, *H*_3_, *H*_4_, *H*_5_, *E*_*k*_, *D*_*k*_〉 to *F* as the public parameters. When receiving the parameter, *F* outputs target multiple identities $\left(ID_{1}^{*},ID_{2}^{*},\cdots ,ID_{n}^{*}\right)$.

**Attack:** *F* does several queries to *B*. These queries are the same as those in Phase 1 of Theorem 4.

**Forgery:** The forger *F* outputs a new ciphertext *C* = 〈*Y*, *U*, *X*, *Z*, *V*, *W*, *a*_0_, *a*_1_, ⋯, *a*_*n*−1_〉. If the forgery succeeds, the equation $e\left(W^{*},P\right)=e\left(X^{*}+h\cdot \gamma Q_{S}^{*},P_{pub}\right)$ holds. Define $Q_{S}^{*}=l_{S}^{*}P=aP$, then compute $W^{*}=\left(h+\alpha \right)\gamma D_{S}^{*}=\left(h+\alpha \right)l_{S}^{*}bP=\left(h+\alpha \right)abP$. Now, we will easily get the solution of CDH problem: *abP* = W*(*α* + *h*)^−1^.

Here, we consider the advantage of *F*’s success. For *q*_*s*_ queries to the Anony-signcrypt queries, the probability for *B* to answer a failure Anony-signcrypt query is less than *q*_*s*_/2^*k*^. So, if the forger *F* wins the game, *B*’s advantage is *ε*′ ≥ *ε* − *q*_*s*_/2^*k*^.

**Theorem 6:** If an ANON-IND-sMID-CCA attacker *A* has a non-negligible advantage *ε* to win the game defined in Definition 6 within running time *t*, then the Gap-BDH problem can be solved by the challenger *B* with a non-negligible advantage *ε*′ ≥ (*ε* − *q*_*d*_/2^*l*^)/*nq*_*H*_2__, where (*q*_*ε*_, *q*_*d*_, *q*_*H*_1__, *q*_*H*_2__, *q*_*H*_3__, *q*_*H*_4__, *q*_*H*_5__) denote the number of Key extract queries, De-signcrypt queries, queries to the hash functions *H*_1_, *H*_2_, *H*_3_, *H*_4_, *H*_5_, respectively. And the running time in which the scheme needs to execute is *t*′ ≈ *t* + (*q*_*ε*_ + *q*_*H*_1__)*O*(*t*_1_) + (*q*_*H*_2__ + *q*_*H*_5__)*O*(*t*_2_) + *q*_*d*_*O*(*t*_1_ + *t*_2_) + (*q*_*H*_3__ + *q*_*H*_4__)*O*(1), where *t*_1_ is the time to perform a scalar multiplication in *G*_1_ and *t*_2_ is the time to perform a pairing *e*.

**Proof:** Receiving the instance (*P*, *aP*, *bP*, *cP*) of the Gap-BDH problem, where $a,b,c\in Z_{q}^{*}$ are unknowns, the attacker *A* can make at most *q*_*g*_ queries to compute *e*(*P*, *P*)^*abc*^ by playing the game with challenger *B* as demonstrated in Definition 6. *B* answers every phase of the ANON-IND-sMID-CCA game in the following way:

Suppose that *A* outputs the target identities $\left(ID_{1}^{*},ID_{2}^{*},\cdots ,ID_{n}^{*}\right)$ after receiving the params. When obtaining the identities $\left(ID_{1}^{*},ID_{2}^{*},\cdots ,ID_{n}^{*}\right)$, *B* selects *S* = (*ID*_*β*_1__, *ID*_*β*_2__, ⋯, *ID*_*β*_1__) at random where *S* ⊂ (*ID*_1_, *ID*_2_, ⋯, *ID*_*n*_).

**Setup:** The challenger *B* sets *Q* = *aP*, *P*_*pub*_ = *bP* and sends the *params* ≡ {*q*, *G*_1_, *G*_2_, *e*, *P*, *P*_*pub*_, *H*_1_, *H*_2_, *H*_3_, *H*_4_, *H*_5_, *E*_*k*_(), *D*_*k*_()} to the attacker *A*. When receiving this query with *ID*_*j*_, *B* answers these queries:

***H*_1_-query:**

1. If $ID_{j}\neq ID_{i}^{*},i\in \left\{1,2,\cdots ,n\right\}$, calculate *Q*_*j*_ = *l*_*j*_ *P*; otherwise, calculate *Q*_*j*_ = *l*_*j*_ *Q*, where *l*_*j*_ is an integer.
2. Put it into *H*_1_-list when no (*ID*_*j*_, *l*_*j*_, *Q*_*j*_) exists in *H*_1_-list.
3. *B* returns *Q*_*j*_.

***H*_2_-query:** The challenger *B* examines if (*P*, *Q*_*i*_, *P*_*pub*_, *cP*, *X*_*j*_) uses the DBDH oracle for *i* ∈ [1, *q*_*H*_2__] when he is queried with *X*_*j*_ ∈ *G*_2_ for some *j* = [1, *q*_*H*_2__]. If it exists, *B* shall terminate the game for *e*(*P*, *P*)^*abc*^ equals ${\left(X_{j}\right)}^{l_{i}^{-1}}$. Otherwise, *B* picks a value $x_{j}\in Z_{q}^{*}$ at random and puts a tuple (*X*_*j*_, *x*_*j*_) into the list *L*_2_. Then, the challenger *B* returns *x*_*j*_ to the adversary *A*.

***H*_3_-query:** As an integer *p*_*j*_ is sent to the *H*_3_ oracle where *j* ∈ [1, *q*_*H*_3__], *B* shall pick a string *w*_*j*_ ∈ {0, 1}^*w*^ at random and puts the tuple (*p*_*j*_, *w*_*j*_) into the list *L*_3_. Then, the string *w*_*j*_ is returned to *A* by the challenger *B*.

***H*_4_-query:** When querying for the string *δ*_*j*_ ∈ {0, 1}^*w*^ where *j* ∈ [1, *q*_*H*_4__], *B* shall pick a string *z*_*j*_ ∈ {0, 1}^|*M*|^ at random and puts the tuple (*δ*_*j*_, *z*_*j*_) into the list *L*_4_. Then, the challenger *B* returns the bit string *z*_*j*_ to the attacker *A*.

***H*_5_-query:** Receiving the tuple 〈*X*_*j*_, *U*_*j*_, *Z*_*j*_, *V*_*j*_, *a*_*j*_0__, *a*_*j*_1__, ⋯, *a*_*j*_*n*−1__〉 where *j* ∈ [1, *q*_*H*_5__], *B* picks a value $h_{j}\in Z_{q}^{*}$ at random and puts the tuple 〈*X*_*j*_, *U*_*j*_, *Z*_*j*_, *V*_*j*_, *a*_*j*_0__, *a*_*j*_1__, ⋯, *a*_*j*_*n*−1__, *h*_*j*_〉 into the list *L*_5_. Then, *B* returns *h*_*j*_.

**Phase 1:** Challenger *B* shall answer the Key extract query and De-signcrypt query from attacker *A* as follows:

**Key extract query:** *A* chooses an identity $ID_{j}\neq ID_{i}^{*}$ where *i* ∈ {1, 2} and sends it to challenger *B*, then *B* scans the list *L*_1_ to find if there is the tuple (*ID*_*j*_, *l*_*j*_, *Q*_*j*_) in *L*_1_. If it was, *B* shall calculate *D*_*j*_ = *l*_*j*_ *P*_*pub*_(= *l*_*j*_ *bP* = *bQ*_*j*_). Otherwise, the challenger *B* selects a $l_{j}\in Z_{q}^{*}$ at random, and calculates *Q*_*j*_ = *l*_*j*_ *P* as well as *D*_*j*_ = *l*_*j*_ *P*_*pub*_. At the same time, the challenger *B* puts a tuple (*ID*_*j*_, *l*_*j*_, *Q*_*j*_) into the list *L*_1_. Finally, *B* sends *D*_*j*_ back to the attacker *A*.

**De-signcrypt query:** The attacker *A* queries *B* and send $B(C_{j},ID_{i}^{*})$ where *i* ∈ {1, 2, ⋯, *n*} and *C*_*j*_ = 〈*Y*_*j*_, *U*_*j*_, *X*_*j*_, *Z*_*j*_, *V*_*j*_, *W*_*j*_, *a*_*j*_0__, *a*_*j*_1__, ⋯, *a*_*j*_*n*−1__〉 When receiving the decryption query, *B* executes the following steps:

1. Check the list *L*_5_ to find the tuple 〈*X*_*j*_, *U*_*j*_, *Z*_*j*_, *V*_*j*_, *a*_*j*_0__, *a*_*j*_1__, ⋯, *a*_*j*_*n*−1__〉. If it was found, *B* can get (*Z*_*j*_, *V*_*j*_) from *L*_5_. Otherwise, *B* returns “failure”.
2. Construct the polynomial *f*(*x*) = *a*_*j*_0__ + *a*_*j*_1__ *x* + ⋯+*a*_*j*_*n*−1__ *x*^*n*−1^+*x*^*n*^.
3. Searching the tuple (*ID*_*j*_, *l*_*j*_, *Q*_*j*_) in the list *L*_1_.
4. For *l* = 1, 2, ⋯, *q*_*H*_2__, perform as follows:
  - Search the tuple (*X*_*l*_, *x*_*l*_) from the list *L*_2_.
  - Examine whether (*P*, *Q*_*i*_, *P*_*pub*_, *U*_*j*_, *X*_*j*_) uses the DBDH oracle by verifying the equation *e*(*P*, *P*)^*l*_*j*_*bγ*^ = *X*_*j*_.
  - If the step above is true, calculate *p*_*l*_ = *f*(*x*_*l*_), $\delta_{j}^{\prime }=V_{j}⨁H_{3}\left(p_{i}\right)$, and $M_{j}=D_{H_{4}}\left(\delta_{j}^{\prime }\right)\left(Z_{j}\right)$.
5. Test whether the equation *e*(*W*_*j*_, *P*) = *e*(*X*_*j*_ + *h*_*j*_ *Y*_*j*_, *P*_*pub*_) or the equation *e*(*W*_*j*_, *Q*_*i*_) = *e*(*X*_*j*_ + *h*_*j*_ *Y*_*j*_, *D*_*i*_) holds where *h*_*j*_ = *H*_5_(*X*_*j*_, *U*_*j*_, *Z*_*j*_, *V*_*j*_, *a*_*j*_0__, *a*_*j*_1__, ⋯, *a*_*j*_*n*−1__). If it holds, then return *M*_*j*_ to *A*.
6. Otherwise, *B* sends “failure” to *A*, which means that there is not a valid ciphertext generated following the proposed scheme.

**Challenge:** *A* sends the plaintext *M* to *B*. Then *B* executes the following steps:

1. Select *δ* ∈ {0, 1}^*w*^ at random.
2. Set *U* = *γP* = *cP*.
3. As *i* = 1, 2, ⋯, *n*, *B* shall check the tuples (*ID*_*j*_, *l*_*j*_, *Q*_*j*_) in the list *L*_1_ and compute *υ*_*i*_ = *H*_2_(*e*(*D*_*i*_, *U*)).
4. Choose $p\in Z_{q}^{*}$ at random and structure a polynomial *f*(*x*) as follows:
  $$\begin{array}{ccc} f(x) & = & \prod_{i=1}^{n}\left(x-\upsilon_{i}\right)+p\left(mod\;q\right)\\ & = & a_{0}+a_{1}x+\cdots +a_{n-1}x^{n-1}+x^{n}. \end{array}$$
5. *B* returns the ciphertext C* to *A*.

**Phase2:** *A* shall query challenge *B* like Phase 1 without querying the information of *S* in the Key extract query and C* in De-signcrypt query.

**Guess:** The attacker *A* gives its guess *β*′ ∈ {1, 2, ⋯, *n*}. At the same time, the challenger *B* picks a tuple (*X*_*j*_, *x*_*j*_) at random from the list *L*_2_ where *j* ∈ *β*′, and chooses the tuple (*ID*_*j*_, *l*_*j*_, *Q*_*j*_) from the list *L*_1_. Finally, *B* outputs ${\left(X_{j}\right)}^{l_{2}^{-1}}$ as the solution to the given instance of the Gap-BDH problem.

Here, we shall discuss the advantage of challenger *B*. For answering the De-signcrypt query, the challenger *B* shall check 〈*X*_*j*_, *U*_*j*_, *Z*_*j*_, *V*_*j*_, *a*_*j*_0__, *a*_*j*_1__, ⋯, *a*_*j*_*n*−1__〉 in *L*_5_, and send back “failure” if it is not found. That is to say, the right value of *H*_5_ hash function can be guessed by the attacker *A*. In this case, *B* may fail at the most probability of *q*_*d*_/*q* with *q*_*d*_ queries to the De-signcrypt oracle. In phase Guess, the challenger *B* shall output the right answer *e*(*P*, *P*)^*abc*^ at the least probability of 2/*nq*_*H*_2__, where *q*_*H*_2__ is the time of the *H*_2_ hash oracle query, and *n* is the number of multiple identities. Hence, the Gap-BDH problem can be solved with a non-negligible advantage *ε*′ ≥ (*ε* − *q*_*d*_/2^*l*^)/*nq*_*H*_2__, where *ε* is the non-negligible advantage of attacker *A*. And the required computation time is *t*′ ≈ *t* + (*q*_*ε*_ + *q*_*H*_1__)*O*(*t*_1_) + (*q*_*H*_2__ + *q*_*H*_5__)*O*(*t*_2_) + *q*_*d*_*O*(*t*_1_ + *t*_2_) + (*q*_*H*_3__ + *q*_*H*_4__)*O*(1), for answering queries in the simulation game above.

## Functional comparison and efficiency analysis

In this section, we will evaluate the functional and efficiency comparison of our scheme with the existing schemes.

### Functional comparison

In terms of the funcation, we compare our scheme with some existing schemes in the sender anonymity, receiver anonymity, decryption fairness and public verification, respectively. The comparison is shown in Table 1.

**Table 1:** Comparison of the functions.

Schemes	Sender anonymity	Receiver anonymity	Decryption fairness	Public verification
[15]	No	No	No	No
[17]	No	No	No	No
[20]	No	No	No	No
[26]	Yes(*)	No	No	No
[27]	Yes(*)	No	No	No
[28]	Yes(*)	No	No	No
[29]	Yes(*)	No	No	No
[31]	Yes	No	Yes	Yes
[*Proposed*]	Yes	Yes	Yes	Yes

(*) denotes that the scheme could suffer from the cross-comparison attack and the joint conspiracy attack.

As is shown in Table 1, the schemes [15, 17, 20] cannot protect the sender anonymity. Though the schemes [26–29] can ensure the sender anonymity to some degree, they could suffer from the cross-comparison attack and the joint conspiracy attack for the use of ring signature.

Table 1 shows that the schemes [15, 17, 20, 26–29, 31] cannot reach the receiver anonymity. For the schemes [15, 17, 20, 26–29], the receivers’ identities are stored in the ciphertext in the form of plaintext, which can lead to the leakage of receivers’ privacy. The scheme [31] also cannot realize the receiver anonymity for the use of the Lagrange interpolation polynomial, each authorized receiver can judge whether anyone else is authorized or not. Meanwhile, the schemes [15, 17, 20, 26–29] cannot realize the fair decryption and public verification properties.

As Table 1 shows, our proposed scheme owns these four functions of the sender anonymity, receiver anonymity, decryption fairness, and public verification. The randomized method were used in our scheme, which uses the public key of the sender multiplied by a random value to hide the identity of the sender and avoid the cross-comparison attack and the joint conspiracy attack. In terms of the weakness of the receiver anonymity existed in Lagrange interpolation polynomial, we adopt the new polynomial method which can solve the problem that the authorized receiver can judge the identity of other receivers. So, our scheme simultaneously owns the sender anonymity and the receiver anonymity, which achieves the complete anonymity. In addition, the decryption fairness and public verification properties are also guaranteed in our scheme.

### Efficiency analysis

For the efficiency, we compare our scheme with several existing schemes in terms of computation complexity and ciphertext length from two aspects: signcryption and de-signcryption. The comparison is shown in Tables 2 and 3 respectively, where *E* stands for bilinear pairing operation, *A* stands for the addition operation in *G*_1_, *Mu* stands for the scalar multiplication in *G*_1_, *Ex* stands for the exponentiation in *G*_2_, *H* stands for hash operation in the encryption step, *S* stands for symmetric encryption and *Param* stands for the number of parameters in the ciphertext. In our scheme, the operation of the polynomial can be pre-processed, so these operations are excluded when considering computational complexity.

**Table 2:** Comparison of the signcryption efficiency.

Schemes	*E*	*A*	*Mu*	*Ex*	*H*	*S*	*Param*	Ciphertext length
[15]	1	*n* + 1	*n* + 5	1	2	0	10	(*n* + 2)|*G*_1_| + |*G*_2_| + |*M*| + *n*|*ID*|
[17]	2	*n* + 1	*n* + 4	2	2	1	8	(*n* + 2)|*G*_1_| + |*M*| + *n*|*ID*| + |*Z*_*q*_|
[20]	0	*n* + 1	*n* + 3	1	2	0	*n* + 9	3|*G*_1_| + |*M*| + *n*|*ID*|
[26]	0	3*m* + *n* − 2	2*m* + *n* + 2	1	*m* + 2	0	11	(*m* + *n* + 2)|*G*_1_| + |*M*| + (*m* + *n*)|*ID*|
[27]	1	2*m* − 3	2*m* + 2	0	*m* + 2	0	10	2|*G*_1_| + *m*|*G*_2_| + 2|*M*| + *m*|*Z*_*q*_|
[28]	1	4*m* − 2	4*m*	0	*m* + 2	0	10	(*m* + 2)|*G*_1_| + |*M*|
[29]	0	3*m* + *n* − 2	2*m* + *n* + 2	1	*m* + 2	0	11	(*m* + *n* + 2)|*G*_1_| + |*M*| + (*m* + *n*)|*ID*|
[31]	1	2	6	1	2	0	10	(*n* + 4)|*G*_1_| + |*M*|
[*Proposed*]	1	0	5	0	*n* + 3	1	13	4|*G*_1_| + |*M*| + *w* + *nZ*_*q*_

|*G*_1_|: the length of the elements in *G*_1_; |*Z*_*q*_|: the length of the elements in *Z*_*q*_; |*ID*|: the length of identity information; |*M*|: the length of the plaintext *M*; *m*: the number of senders; *n*: the number of receivers; *w*: the bit length of a string

**Table 3:** Comparison of the signcryption efficiency.

Schemes	Public verification	Judgment	Decryption
[15]	3*E* + 2*A* + 3*Mu* + 3*H*	3*E* + 2*A* + 3*Mu* + 3*H*	3*E* + 2*A* + 3*Mu* + 3*H*
[17]	2*E* + *Ex* + *Mu* + 2*H*	2*E* + *Ex* + *Mu* + 2*H*	4*E* + 2*T*_*a*_ + *Ex* + 3*H* + *T*_*s*_
[20]	3*E* + 2*A* + (3*n* + 3)*Mu* + 2*Ex* + (*n* + 1)*H*	3*E* + 2*A* + (3*n* + 3)*Mu* + 2*Ex* + (*n* + 1)*H*	3*E* + 2*A* + (3*n* + 3)*Mu* + 2*Ex* + (*n* + 1)*H*
[25]	2*E* + *A* + *Mu* + *H*	2*E* + *A* + *Mu* + *H*	2*E* + *nA* + (*n* − 1)*Mu* + 2*H*
[26]	2*E* + (2*m* − 1)*T*_*a*_ + *Mu* + *mH*	4*E* + 2*mA* + (*m* + 1)*Mu* + (*m* + 1)*H*	4*E* + 2*mA* + (*m* + 1)*Mu* + (*m* + 1)*H*
[27]	3*E* + (*m* + 1)*T*_*a*_ + 2*mMu* + (*m* + 2)*H*	*N*/*A*	3*E* + (*m* + 1)*T*_*a*_ + 2*mMu* + (*m* + 2)*H*
[28]	4*E* + 2*mT*_*a*_ + *mT*_*m*_ + (*m* + 2)*H*	*N*/*A*	4*E* + 2*mT*_*a*_ + *mMu* + (*m* + 2)*H*
[29]	(*M* + 5)*E* + *A* + (*m* + |*M*| + 2)*Mu* + 2*H*	(*M* + 5)*E* + *A* + (*m* + |*M*| + 2)*Mu* + 2*H*	(*M* + 5)*E* + *A* + (*m* + |*M*| + 2)*Mu* + 2*H*
[31]	2*E* + *A* + *Mu* + *H*	2*E* + *A* + *Mu* + *H*	2*E* + *nA* + (*n* − 1)*Mu* + 2*H*
[*Proposed*]	2*E* + *A* + *Mu* + *H*	2*E* + *A* + *Mu* + *H*	*E* + *S* + 3*H*

|*M*|: the length of the plaintext *M*; *m*: the number of senders; *n*: the number of receivers.

As is shown in Table 2, we can see that our proposed scheme used one bilinear pairing operation *E*. Though the bilinear pairing operation has high cost, our scheme controls it within acceptable limits by comparing with others. In terms of hash operation, because of lower cost than other operation, it is within acceptable limits. Encryption algorithm *S* is used in our scheme, which can be chosen according to practical applications. So, it is easy to reasonably control its communication cost. Meanwhile, our scheme has obvious improvement in operation *A*, scalar multiplication, exponentiation and ciphertext operation. It can be seen that our scheme has better efficiency in signcryption.

On the other hand, in the de-signcryption process, there are generally three algorithms affecting the efficiency: public verification, judgment, and decryption. We will compare the proposed scheme with the existing schemes about these three algorithms, respectively.

As shown in Table 3, our scheme and sheme [31] have obviously higer efficiency in public verification and authorization judgement comparing with the other schemes [15, 17, 20, 26–29], where N/A indicates that the scheme only considered the single receiver environment, which is tansfered via unicast channel. In this case, it is unnecessary to judge whether the receiver is authorized or not. Meanwhile, our scheme has higher efficiency than others in decryption.

From the above analysis, though our scheme has unobvious improvement on the efficiency in general, it owns the complete anonymity containing the sender and receiver anonymity, which is an excellent contribution we think. In our scheme, any receiver can only judge whether the ciphertext is from a reliable sender or not, rather than actually getting the real identity of the sender. Attackers not only outside the system but also inside the system can be prevented in our new scheme.

Besides the above theoretical analysis on efficiency, we shall also give some experiment results to compare our scheme with the existing ones more intuitively. Like the work [35–37], we shall also pay attention to those time-consuming operations and overlook the other ones that do not consume much time. We define the following notations in Table 4, and borrow the experiment testing results from [35–37].

**Table 4:** Notation and definition of diffident time complexities.

Notations	Definition and conversion
*T*_*M*_	Time required for executing a modular multiplication operation.
*T*_*E*_	Time required for executing a bilinear pairing operation, *T*_*E*_ ≈ 87*T*_*M*_.
*T*_*A*_	Time required for executing a point addition of two points in *G*_1_, *T*_*A*_ ≈ 0.12*T*_*M*_.
*T*_*Mul*_	Time required for executing a scalar multiplication in *G*_1_, *T*_*Mul*_ ≈ 29*T*_*M*_.
*T*_*Exp*_	Time required for executing a exponentiation in *G*_2_, *T*_*Exp*_ ≈ 43.5*T*_*M*_.
*T*_*H*_	Time required for executing a hash operation, *T*_*H*_ ≈ 29*T*_*m*_.

Then, with the results in Table 4, the efficiency comparison of our scheme with the existing ones can be shown by Tables 5 and 6.

**Table 5:** Time complexity comparison of signcryption.

Schemes	Time complexity of signcryption
[15]	(29.12*n* + 333.62)*T*_*M*_
[17]	(29.12*n* + 435.62)*T*_*M*_
[20]	(29.12*n* + 188.62)*T*_*M*_
[26]	(87.36*m* + 29.12*n* + 159.26)*T*_*M*_
[27]	(87.24*m* + 192.64)*T*_*M*_
[28]	(145.48*m* + 144.76)*T*_*M*_
[29]	(87.36*m* + 29.12*n* + 159.26)*T*_*M*_
[31]	362.74*T*_*M*_
*Proposed*	(29*n* + 319)*T*_*M*_

**Table 6:** Time complexity comparison of de-signcryption.

Schemes	Public verification	Judgment	Decryption
[15]	435.24*T*_*M*_	435.24*T*_*M*_	435.24*T*_*M*_
[17]	304.5*T*_*M*_	304.5*T*_*M*_	478.14*T*_*M*_
[20]	(116*n* + 464.24)*T*_*M*_	(116*n* + 464.24)*T*_*M*_	(116*n* + 464.24)*T*_*M*_
[26]	(58.24*m* + 173.88)*T*_*M*_	(58.24*m* + 406)*T*_*M*_	(58.24*m* + 406)*T*_*M*_
[27]	(87.12*m* + 319.12)*T*_*M*_	N/A	(87.12*m* + 319.12)*T*_*M*_
[28]	(58.24*m* + 406)*T*_*M*_	N/A	(58.24*m* + 406)*T*_*M*_
[29]	(116*m* + |*M*|*m* + 117.12)*T*_*M*_	(116*m* + |*M*|*m* + 117.12)*T*_*M*_	(116*m* + |*M*|*m* + 117.12)*T*_*M*_
[31]	232.12*T*_*M*_	232.12*T*_*M*_	(29.12*n* + 203)*T*_*M*_
*Proposed*	232.12*T*_*M*_	232.12*T*_*M*_	174*T*_*M*_

|*M*|: the length of the plaintext *M*; *m*: the number of senders; *n*: the number of receivers.

Tables 5 and 6 also show the relative high efficiency of our scheme when compared with the exiting schemes with the same functions.

## Conclusion

A novel multi-receiver signcryption scheme with complete anonymity is proposed in this paper. By using a new polynomial technology, our scheme actually achieves the receiver anonymity. Attackers not only outside the system but also inside the system can be prevented in our new scheme. Meanwhile, in the process of signcryption, the sender used the randomized method to hide its public key, which ensures the sender anonymity. So, our scheme simultaneously owns the sender anonymity and the receiver anonymity, which achieves the complete anonymity. In addition, the decryption fairness and public verification properties are guaranteed in our scheme. This new scheme can be applied better to secure broadcast, network meeting, paying-TV and data sharing on the cloud.
